# *COMT* and *GAD1* gene polymorphisms are associated with impaired antisaccade task performance in schizophrenic patients

**DOI:** 10.1007/s00406-018-0881-7

**Published:** 2018-02-10

**Authors:** Anna V. Kirenskaya, Zinaida I. Storozheva, Marina A. Gruden, Robert D. E. Sewell

**Affiliations:** 1 Federal Medical Research Centre of Psychiatry and Narcology, Kropotkinsky Lane. 23, 119034 Moscow, Russian Federation; 2 Federal State Budgetary Scientific Institution “P. K. Anokhin Research Institute of Normal Physiology”, Baltiskaya St., 8, 125315 Moscow, Russian Federation; 3 Cardiff School of Pharmacy and Pharmaceutical Sciences, Redwood Building, Cardiff University, Cardiff, CF10 3NB UK

**Keywords:** *COMT rs4680* polymorphism, *GAD1 rs3749034* polymorphism, Antisaccade task, Schizophrenia

## Abstract

Genetic influences modulating executive functions engaging prefrontal cortical brain systems were investigated in 141 male subjects. The effects of variations in two genes implicated in dopamine and GABA activities in the prefrontal cortex: *rs4680* (Val158/Met polymorphism of the catechol-*o*-methyltransferase gene—*COMT*) and *rs3749034* (C/T) substitution in the promoter region of the glutamic acid decarboxylase gene (*GAD1*) were studied on antisaccade (AS) performance in healthy subjects and schizophrenic patients. Genotyping revealed a trend towards a reduced proportion of *COMT* Val/Met heterozygotes and a significantly increased frequency of the *GAD1 rs3749034* C allele in schizophrenic patients relative to controls. Patients had elevated error rates, increased AS latencies and increased latency variability (coefficient of variation) compared to controls. The influence of polymorphisms was observed only in patients but not in controls. A substantial effect of the *COMT* genotype was noted on the coefficient of variation in latency, and this measure was higher in Val homozygotes compared to Met allele carriers (*p* < 0.05) in the patient group. The outcome from *rs3749034* was also disclosed on the error rate (higher in T carriers relative to C homozygotes, *p* < 0.01) and latency (increased in C homozygotes relative to T carriers, *p* < 0.01). Binary logistic regression showed that inclusion of the genotype factor (i.e., selective estimation of antisaccade measures in CC carriers) considerably increased the validity of the diagnostic model based on the AS measures. These findings may well be derived from specific genetic associations with prefrontal cortex functioning in schizophrenia.

## Introduction

There are numerous reports from structural and neuroimaging studies of disturbed prefrontal cortical (PFC) function in schizophrenia [1–3]. Impaired performance in complex tasks critically engaging prefrontal cortical systems requiring advanced cognitive processes are collectively known as executive functions and they are an important component underlying many of the cognitive deficits observed in schizophrenia [1, 4, 5]. In the context of this, it has been suggested that cognitive deficits related to executive functions are particularly heritable [6, 7].

The genetic contribution to the etiology of schizophrenia is considerable, and recent molecular genetic studies indicate that several genes may be associated with the disorder. In an endeavor to expose susceptibility genes, schizophrenia research has focused on an endophenotypic approach. Endophenotypes are relatively simple, distinct and measurable biobehavioral characteristics. Individually, they are intermediary between the cellular effects of susceptibility genes manifesting psychopathology and they are undoubtedly determined by fewer genes than the more complex phenotype of schizophrenia [1, 8–11]. The antisaccade (AS) task has been employed as an endophenotype, and performance impairment in this task is considered an indicator of genetic propensity towards fronto-striatal pathologies such as schizophrenia [12].

AS performance involves a range of executive processes that rely heavily on frontal lobe structures including directed attention and inhibition, task management, planning, monitoring, and coding of representations in working memory [13, 14]. The importance of frontal cortical regions in AS performance has been established using functional brain imaging [15–19], and examination of event-related cortical potentials [20–22]. Increased neural firing in the prefrontal cortex before antisaccades has also been demonstrated in several studies in non-human primates [23–25].

The performance deficit exhibited by schizophrenic patients in the AS task has been interpreted as support for prefrontal cortical dysfunction and abnormalities in several related prefronto-centric circuits. It is notable that antisaccade deficits occur in unaffected family members of schizophrenic patients, and the large difference in effect size between schizophrenia probands, relatives and normal healthy controls renders the antisaccade task highly appropriate for endophenotype genetic studies [12, 26]. However, only a limited number of investigations have been carried out in this regard [27–30].

Numerous evidence-based treatments for psychosis point to the dopaminergic and other neurotransmitter systems, upon which much supportive scientific data has accumulated. In relation to this, an association between genes linked to dopamine function and schizophrenia has been widely established in candidate-gene studies [1, 9–11, 31]. However, genome-wide association studies (GWAS) have confirmed relatively few of these associations though a post-GWAS has identified not only *COMT* but also *GAD1* among a selection of schizophrenia risk genes [32]. There can be discrepancy between the results of pre-GWAS (candidate-gene) and GWAS studies with respect to disease. Consequently, an omnigenic model has been proposed in which assorted genes expressed in disease-relevant cells may perturb the functions of core disease-related genes [33] and this concept is likely to be the focus of future debate. In the case of schizophrenia, the dopaminergic pathways are one of the main targets of treatment, and genes participating in the regulation of dopaminergic neurotransmission are among the most relevant hypothesis-driven candidates. Since hypofrontality greatly contributes to cognitive deficit in schizophrenia, the study of genes linked to prefrontal dopamine activity contributes towards the understanding of the neurophysiologic and genetic mechanisms of the disease.

The catechol-*O*-methyltransferase gene (*COMT*) produces an enzyme that degrades catecholamines and is known to be one of the key factors in the regulation of dopamine level in the prefrontal cortex (PFC) [34]. *COMT* is among the so-called “hypothesis-driven” candidate genes for the risk of schizophrenia [32]. A Val158Met single nucleotide polymorphism of the *COMT* gene (*rs4680*) influences its enzyme activity. Thus, the Met/Met variant displays 40% less enzymatic activity than the Val/Val variant [35], and is consequently associated with higher dopamine levels in the prefrontal and anterior cingulate cortex [36]. The *COMT* Val158Met polymorphism correspondingly affects several cognitive functions including attention and executive control [37]. It has also been found that the Val/Val genotype is associated with smaller grey matter density in the anterior cingulate cortex [38] hippocampus, amygdala-uncus and middle temporal gyrus [39].

A dopamine-GABA interplay in the prefrontal cortex sustains working memory and attention [40]. Moreover, schizophrenia is associated with alterations in several markers of GABA neurotransmission in multiple cortical regions, including the prefrontal, primary motor, primary visual, and anterior cingulate cortices [41, 42–45]. GABAergic activity strongly depends on glutamic acid decarboxylase (GAD67), the enzyme that metabolizes glutamate to GABA and is responsible for the production of the majority of GABA in the brain [45]. Like *COMT*, the *GAD1*gene that encodes GAD67 is a hypothesis-driven gene-candidate for the risk of schizophrenia [32]. Downregulation of GAD67 in the fast-spiking parvalbumin-positive interneurons of the dorsolateral prefrontal cortex (DLFPC) is an authenticated result in postmortem schizophrenics [46, 47], though this finding has not yet been followed up in genetic studies. The polymorphism *rs3749034* (C/T substitution in the 5′-untranslated region in intron 1) is one of the few polymorphisms of *GAD1* with identified functional consequence. The C-allele of the polymorphism *rs3749034* is associated with a decreased level of transcription of the enzyme, a genetic risk for childhood onset of schizophrenia and decreased cortical thickness [48–50]. More recently, a Т-allele association with lower white matter fractional anisotropy (FA) has been demonstrated, as well as the effects of lower white matter FA on poor working memory performance and frontal executive functioning that are independent of diagnosis [51].

Despite the fact that a number of investigations concerning associations between *COMT* and *GAD1* genes with schizophrenia have been performed, the conclusions from these reports are somewhat open to debate. The majority of studies using intermediate phenotypes have examined the influence of a single genetic variant on brain activity and behavior. More recent work suggests that considering the effects of a gene in the context of additional and interacting factors may further elucidate gene–brain–behavior relationships.

The approach that may provide a more comprehensive understanding of the phenotypic effects of genetic variations is the study of gene-trait (G x T) interaction [52]. A trait may indicate a psychological characteristic or the presence or absence of a diagnosis in research on psychopathology. It may also describe a variation in the general organismic context in which any single gene operates. Emerging from this, it is important that studies directly comparing genetic influences on neurophysiological phenotypes are performed in both healthy persons and schizophrenic patients. This approach may also be fruitful for developing diagnostic models that include genetic and neurophysiological data, although such studies are scarce.

To study the role of genetic influences in modulating behavior during cognitive control related to PFC networks, we investigated the effects of polymorphisms of *COMT* and *GAD1* genes on antisaccade performance in healthy controls versus schizophrenic patients. Bearing in mind that sexual dimorphism is a phenotypic consequence of genes [53], only male subjects were recruited to the study. Dominant handedness is also an important human characteristic tendency and relationships between handedness and personality traits, cognitive styles, as well as psychopathology have been established. There is also evidence of lateralized activity in DA neurotransmitter pathways in the human brain [54]. Thus, an interaction between handedness and genetic variants can be assumed, therefore only right-handed subjects were selected for the study.

Additionally, taking into account the genotypes studied, we assessed the possibility of using AS data as a predictor in a diagnostic model for schizophrenia.

## Experimental procedures

### Participants

A total of 141 male subjects (aged 19–54 years) participated in the study. Both schizophrenia patients (SCH group, *n* = 78) and healthy volunteer controls (CON group, *n* = 63) were recruited and all participants were right-handed males.

The patients (mean age 31.8 ± 1.2 years) were admitted to the Serbsky National Centre for Social and Forensic Psychiatry to undergo examination: 75 patients were criminal offenders, and 3 individuals were examined within the framework of civil litigation. Most of the patients (63 subjects) were classified as paranoid (F20.06), 7 patients had undifferentiated schizophrenia (F20.3), 3 patients had residual schizophrenia (F20.5) and 4 patients had simple schizophrenia (F20.6) according to ICD-10. Current patient symptomatology was assessed using the Positive and Negative Symptom Scale (PANSS). The mean sum of PANSS scores was 85.36 ± 1.50.

None of the patients displayed acute symptoms and they were free from medication for a period of approximately one month before investigation and prior to initiation of drug treatment. Healthy participants (63 subjects, mean age 25.8 ± 0.55 years) were evaluated by a psychiatrist to exclude any psychiatric diagnosis. Individuals with a history of neurological disorder, head trauma with loss of consciousness, substance abuse, or other medical conditions that might conceivably affect brain functioning were excluded from participation in the study. After a detailed description of the study, written informed consent for the investigation was obtained from all subjects in accordance with the local ethics committee of the Federal Medical Research Centre of Psychiatry and Narcology, Moscow, Russian Federation and the study was performed in accordance with the ethical standards laid down in the 1964 Declaration of Helsinki.

### Genotyping

Extraction of DNA from saliva samples was done with MagMAX^™^ DNA Multi-Sample Ultra Kits (Applied Biosystems Cat No A25597). Determination of both *COMT* Val158Met and *GAD1 rs3749034* polymorphisms was performed using real-time polymerase chain reaction using TaqMan^®^ SNP Genotyping Assays C__25746809_50 for rs4680 and C__2177452_1 for *rs3749034* (Applied Biosystems, USA) with LightCycler® Nano Instrument, Roche Molecular Systems Inc, USA.

### Antisaccade study procedure

**Fig. 1:**
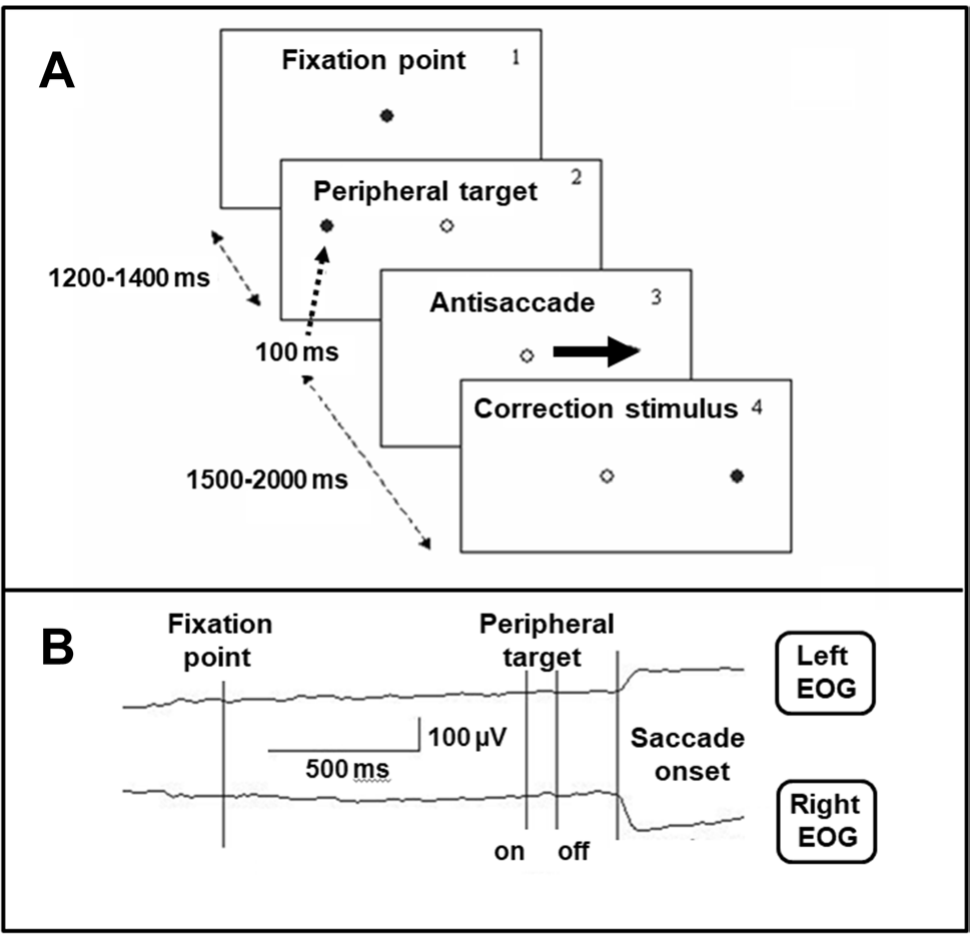
Antisaccade experimental paradigm and examples of Electrooculogram (EOG) recording. **a** Experimental design of the visual stimuli presentation (dark circle—stimulus switched “on”; light circle—stimulus switched “off”). **b** Example of EOG recording

Experiments were carried out in a darkened room. The subjects were seated 100 cm in front of a board with 3 red light-emitting diodes 5 × 5 mm (LEDs) (one central and two peripheral LEDs located 10° to the left and right of one positioned centrally). The central LED was used as the fixation point (FP) and the two other LEDs were used as peripheral targets (PT) (Fig. 1, panel 1 and 2). Participants performed antisaccades and were instructed to look as quickly as possible at the horizontal mirror position of the PT (Fig. 1, panel 3). During the experiment, the FP was extinguished simultaneously with PT onset. The duration of the interval between the FP and PT onset varied between 1200 and 1400 ms and the PT duration was set at 100 ms. Left or right target locations were randomly chosen for each trial. A correction visual stimulus at the mirror position was presented 1.5–2.0 s after the PT onset (Fig. 1, panel 4). To initiate each trial, the subject pressed and held down a mouse button with the right index finger, and FP was switched on 100–1500 ms after the press. The mouse was located on an elbow-rest for convenience. The button press was introduced in the experimental paradigm to increase attention level and behavioral engagement in the task, because these factors are generally reduced in schizophrenic patients. The LEDs were controlled by a custom-designed computer program and all intervals were varied randomly. During every experiment, 3–4 blocks each consisting of 45 trials was presented. Most frequently, 3 blocks (135 trials in total) were presented; 4 blocks were presented in the cases of patients displaying poor task performance. The rest interval imposed between the blocks lasted 3 min and a short pre-training session was introduced before the main experiment.

### Recording and data analysis

A horizontal electrooculogram (EOG) was recorded via skin electrodes placed at the outer canthi of both eyes with the left earlobe as recording reference using an EEG-24 AC amplifier (MBN, Russia). The EOG channel was used for automatic saccade onset detection and for measuring both saccade latency and direction. Saccade onset was defined as a starting point of EOG deflection exceeding a predetermined threshold by slope, and where the amplitude exceeded an EOG standard variation value. The sign of deflection determined the saccade direction and each individual trial was classified by means of latency and direction as being correct or incorrect. Regular saccades only (with latency > 120 ms) [13] were included in the analysis. The following performance indices were obtained:

(1) antisaccade reflexive error rate which reflected the percentage of error trials over the total number of valid trials, (2) latency of correct antisaccades which was defined as the time (ms) from target to saccade onset, (3) coefficient of variation of the latency (CV, %) for correct saccades as a measure of intra-individual latency variability.

### Adjustment of groups for age

Age was differentially distributed across groups. Accordingly, the mean age of the schizophrenic patients was somewhat greater than the healthy controls (31.8 ± 1.1 vs. 25.8 ± 0.55 years), (*F* [1, 139] = 24.3, *p* < 0.001). Consequently, to avoid any false conclusions caused by an influence of age on the parameters studied, groups were adjusted for age by exclusion of the youngest 15% of healthy controls and the oldest 15% of patients [55].

Age adjustment was performed for analysis of antisaccade measures and PANSS scores, while between-group differences in genotypes were analyzed for all participants.

### Statistical analysis

Statistical analysis of results was performed using STATISTICA 6.0 software. The Chi square test was used to evaluate the Hardy–Weinberg genotypic frequencies and the Fisher exact test was used to evaluate the proportion of different alleles of *rs4680* and *rs3749034* in healthy control (CON) and schizophrenia patient (SCH) groups.

Two-way ANOVA (General Linear Model procedure, GLM) was applied to study the effects of group, genotype and their interaction on the measures of the antisaccade task performance. Post hoc comparisons were performed using Student’s t-test with Bonferroni correction for multiple comparisons. Spearman’s correlations between PANSS scores and AS measures were analyzed separately for different *COMT* and *GAD1* genotypes.

## Results

### Genotyping: distribution of *COMT* and *GAD1* polymorphisms in control and schizophrenic patient groups

#### *COMT rs4680* polymorphism schizophrenic patient and control group distribution outcomes

The distribution of the *COMT* genotype in the control group was within the Hardy–Weinberg equilibrium (Table 1). However, a significant deviation from the Hardy–Weinberg equilibrium equation was observed in the clinical patient group.

**Table 1:** Genotype frequencies of *rs4680* (*COMT*) and *rs3749034* (*GAD*1) in the control (Con) and schizophrenic patient (Sch) groups

SNPs	Group	Frequencies % (n)					*p* (*χ* ^2^)*
		Alleles		Genotypes			
rs4680		Val	Met	Val/Val	Val/Met	Met/Met	
	Sch (*n* = 78)	43.0% (80)	57.0% (106)	32.0% (25)	38.5% (30)	29.4% (23)	*P* = 0.024 (*χ* ^2^ = 4.81)
	Con (*n* = 63)	51.6% (65)	48.4% (61)	27.0% (17)	49.2% (31)	23.8% (15)	*P* = 0.125 (*χ* ^2^ = 2.35)
rs3749034		C	T	C/C	C/T	T/T	
	Sch (*n* = 78)	73.1 (114)#	26.9 (42)	59.8% (42)	38.5% (30)	7.7% (6)	*P* = 0.365 (*χ* ^2^ = 0.81)
	Con (*n* = 63)	62.7% (79)	37.3% (47)	38.1% (24)	49.2% (31)	12.7% (8)	*P* = 0.680 (*χ* ^2^ = 0.17)

*p deviation from the Hardy–Weinberg equilibrium distribution

*#p* < 0.05 relative to Con group

Comparison of genotype frequencies within the control group revealed that the proportion of Val/Met heterozygotes was significantly higher than either Val homozygotes (*p* = 0.011) or Met homozygotes (*p* = 0.002). Alongside this, the relative proportions of various genotypes did not vary (*p* > 0.1) within the patient group.

#### *GAD1 rs3749034* polymorphism schizophrenic patient and control group distribution outcomes

In both groups, the distribution of the *GAD1* genotype was within the Hardy–Weinberg equilibrium (Table 1). The frequency of the C allele in the schizophrenic patient group was significantly higher than it was in the healthy controls (73.1% compared to 62.7%, *p* = 0.038).

### Absence of any age or educational level influence on *COMT* and *GAD1* genotype distribution in schizophrenic patient and control groups

After adjustment of groups for age and artifact rejection, 65 patients and 53 healthy subjects were included in the analysis of antisaccade measures and PANSS scores. The mean age in the control group was 26.4 ± 1.1 years and the mean age in the schizophrenic patient group was 27.1 ± 1.3 years [*F*(1,116) = 1.15, *p* = 0.32]. Demographic characteristics of included participants from both groups in relation to their *COMT* and *GAD1* genotype are presented in Table 2. No significant age differences existed between *COMT* and *GAD1* genotypes in either the patient or control groups and no group x genotype interaction was observed (*F* < 1.18, *p* > 0.38). Education level was significantly higher in the control group (14.9 ± 0.3 years) relative to the schizophrenic group (12.4 ± 0.3 years) [*F* (1,116) = 22.2, *p* < 0.001]. However, no effect of *COMT* or *GAD1* genotype on education was observed, neither was there any group x genotype interaction (*F* < 1.6, *p* > 0.21).

**Table 2:** Demographic characteristics of heathy control subjects and schizophrenic patients (included in the antisaccade analysis) in relation to their total and *COMT* and *GAD1* genotypes

Parameters	Group	Polymorphism	Genotype	Mean ± SEM
Age (years)	Healthy control (*n* = 53)	*rs4680* (*COMT*)	Met/Met (*n* = 13)	26.2 ± 1.6
			Val/Met (*n* = 26)	25.9 ± 1.4
			Val/Val (*n* = 14)	27.5 ± 1.1
		*rs3749034* (*GAD1*)	C/C	27.3 ± 1.4
			C/T + T/T	25.7 ± 1.2
	Schizophrenia patients (*n* = 65)	*rs4680* (*COMT*)	Met/Met (*n* = 20)	25.8 ± 1.4
			Val/Met (*n* = 24)	27.3 ± 1.2
			Val/Val (*n* = 21)	28.6 ± 1.8
		*rs3749034* (*GAD1*)	C/C	27.1 ± 1.0
			C/T + T/T	26.8 ± 1.4
Education (years)	Healthy control (*n* = 53)	*rs4680* (COMT)	Met/Met (*n* = 13)	15.1 ± 0.4
			Val/Met (*n* = 26)	14.6 ± 0.3
			Val/Val (*n* = 14)	15.0 ± 0.4
		*rs3749034* (*GAD1*)	C/C	15.1 0.5
			C/T + T/T	14.8 0.3
	Schizophrenia patients (*n* = 65)	*rs4680* (*COMT*)	Met/Met (*n* = 20)	11.2 ± 0.3
			Val/Met (*n* = 24)	12.9 ± 0.9
			Val/Val (*n* = 21)	12.3 ± 0.6
		*rs3749034* (GAD1)	C/C	13.0 0.4
			C/T + T/T	11.8 0.4

### PANSS scores for genotypes in schizophrenic patients

The clinical characteristics of the patients across genotypes are presented in Table 3. ANOVA revealed a significant effect [*F* (2,70) = 3.84, *p* < 0.05] of the *COMT* genotype on the General Psychopathology Scores (PANSS). The Val/Val carriers had higher General Psychopathology Scores (PANSS) than either the Met/Met (*p* = 0.043) or Val/Met (*p* = 0.029) variants. Other effects of studied genotypes were insignificant.

**Table 3:** Symptom ratings (PANSS; mean ± SEM) in schizophrenic patients (included in the antisaccade analysis) in relation to their *COMT* and *GAD1* genotypes

Sample	PANSS total score	PANSS positive score	PANSS negative score	PANSS general score
Total	85.5 ± 1.8	19.4 ± 0.7	22.4 ± 0.7	43.9 ± 1.0
Carriers of the Met/Met variant of *COMT* (*n* = 20)	84.7 ± 2.4	19.3 ± 1.2	22.1 ± 1.3	42.9 ± 1.7
Carriers of the Val/Met variant of *COMT* (*n* = 24)	82.0 ± 2.9	18.1 ± 1.2	22.1 ± 1.4	42.1 ± 1.8
Carriers of the Val/Val variant of *COMT* (*n* = 21)	91.2 ± 3.5	21.1 ± 1.4	22.6 ± 1.4	47.9 ± 2.0* #
Carriers of the C/C variant of *GAD1* (*n* = 34)	87.1 ± 2.2	19.8 ± 0.9	23.3 ± 0.9	44.0 ± 1.2
Carriers of the T allele of *GAD1* (*n* = 31)	83.8 ± 2.0	19.4 ± 0.9	21.6 ± 0.9	43.8 ± 1.2

*p < 0.05 relative to carriers of the Val/Met variant of COMT

#*p* < 0.05 relative to carriers of the Met/Met variant of COMT

### Genotypes and antisaccade task performance

#### *COMT* genotype and antisaccades

**Fig. 2:**
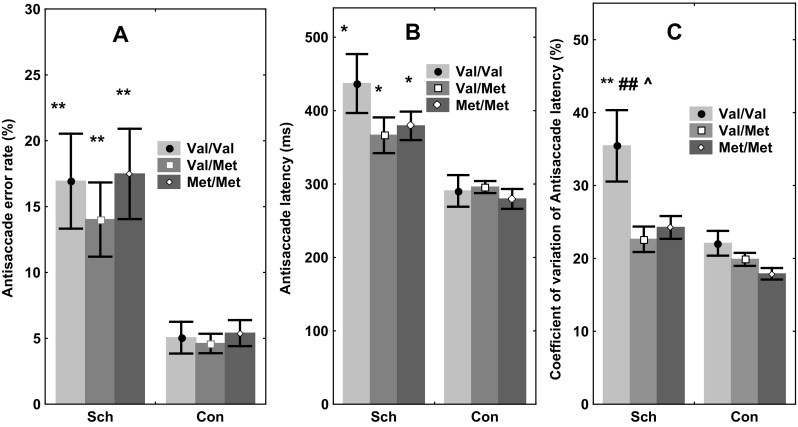
The effects of *COMT* Val158Met polymorphism on antisaccade parameters in schizophrenic patients (Sch) and healthy controls (Con). **a** Antisaccade error rate (%), **p < 0.01 relative to the carriers of the same *COMT* genotype from the Con group. **b** Antisaccade latency (ms), *p < 0.05 relative to the carriers of the same *COMT* genotype from the Con group. **c** Coefficient of variation of antisaccade latency, (%). **p < 0.01 relative to the carriers of the Val/Val *COMT* genotype from the Con group, ^##^ *p* < 0.01 relative to Val/Met carriers from the Sch group, ^^^ *p* < 0.05 relative to Met/Met carriers from Sch group

To test the hypothesis that *COMT* Val158Met affected the measures of antisaccade task performance, a GLM ANOVA analysis was performed. There was no significant effect of genotype on the error rate [*F*(2, 114) = 0.41, *p* = 0.6] in the pooled sample of patients and controls. In addition, no genotype x diagnosis interaction was exposed [*F*(1,2, 112) = 0.389, *p* = 0.678], though there was a highly significant effect of diagnosis on the error rate [*F*(1, 116) = 19.02, *p* = 0.00004] (Fig. 2a).

Additionally, comparison of Val carriers (Val/Val + Val/Met genotypes) versus Met homozygotes, or Met carriers (Met/Met + Val/Met genotypes) versus Val homozygotes did not disclose any association between *COMT* genotype and error rate.

Similar outcomes were obtained for the latency of correct saccades. Hence, there was neither a significant effect of genotype [*F*(2, 114) = 0.98, *p* = 0.39] nor any interaction between genotype x diagnosis. Nevertheless, the effect of diagnosis on saccade latency was once again highly significant [*F*(1, 116) = 18.6, *p* < 0.001], although comparison of Val carriers versus Met homozygotes, or Met carriers versus Val homozygotes did not divulge any influence of *COMT* genotypes on saccade latency (Fig. 2b).

In relation to the coefficient of variation (CV) for the latency of correct saccades, a significant effect of genotype [*F*(2, 114) = 6.1, *p* < 0.01] was disclosed in the pooled sample of patients and controls. No significant genotype x diagnosis interaction was recorded [*F*(1, 2, 112) = 2.41, p = 0.076]. However, the effect of diagnosis on saccades was highly significant [*F*(1, 116) = 18.2, *p* < 0.0001] (Fig. 2c).

Furthermore, in the patient group, post hoc *t* test analysis with Bonferroni correction for multiple comparisons unveiled a substantially increased CV in the Val homozygote sample compared to either Val/Met heterozygotes (*p* = 0.009) or Met homozygotes (*p* = 0.015). On the other hand, there was no effect of *COMT* genotype on the CV in the control group (*p* > 0.21).

Analysis of CV differences between the samples of Val homozygotes and Met carriers also revealed significant effects for genotype [*F*(2, 114) = 12.3, *p* = 0.001], genotype x diagnosis interaction [F(1, 2, 112) = 5.11, p = 0.021] and diagnosis alone [*F*(1, 116) = 20.4, *p* < 0.001] in the pooled sample. Post hoc comparisons showed distinctly increased CV in the Val homozygotes compared to Met carriers in the patient group (*p* = 0.0005), but not in the controls (*p* = 0.68).

#### *GAD1* genotype and antisaccades

Data from 65 patients (34 C homozygotes, 25 C/T heterozygotes and 6 T homozygotes) in addition to 53 healthy subjects (22 C homozygotes, 24 C/T heterozygotes and 7 T homozygotes) were included in the analysis of antisaccade measures. Due to the low number of T homozygotes, combined samples of T allele carriers (C/T + TT genotypes) and C homozygotes were compared.

**Fig. 3:**
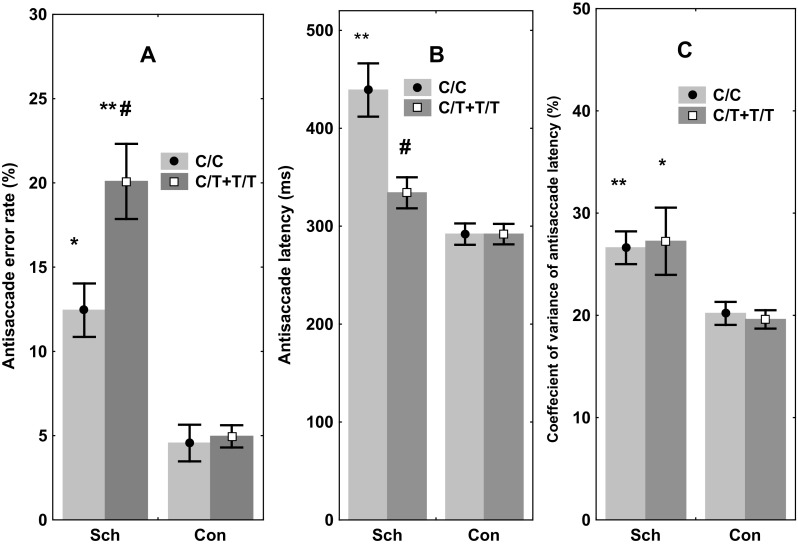
The effects of *rs3749034* polymorphism of *GAD1* on antisaccade parameters in schizophrenic patients (Sch) and healthy controls (Con). **a** Error rate, %. *p < 0.05, **p < 0.01 relative to the carriers of the same genotype from the Con group, ^#^ *p* < 0.05 relative to the carriers of the C/C genotype from Sch group, **b** Latency, ms. **p < 0.01 relative to the cariers of C/C genotype from Con group, ^#^ *p* < 0.05 relative to the carriers of C/C genotype from Sch group. **c** Coefficient of variation of antisaccade latency, %. *p < 0.05, **p < 0.01 relative to the carriers of the same genotype from the Con group

In the case of the error rate, significant effects of genotype [*F*(1, 116) = 5.41, *p* = 0.023] and diagnosis [*F*(1, 116) = 15.23, *p* < 0.001] were observed. Also, post hoc comparisons revealed that mean values of error rate were higher in T carriers compared to C homozygotes, and the differences were significant for the patient group (*p* = 0.023), but not for the healthy controls (*p* = 0.65) (Fig. 3a).

Statistically significant effects of genotype [*F*(1, 116) = 4.10, *p* = 0.020], diagnosis [*F*(1, 116) = 24.87, *p* < 0.001], and genotype x diagnosis interactions [*F*(1,1, 114) = 8.94, *p* < 0.01] were observed to the latency of correct saccades. Additionally, post hoc comparison of means confirmed an increased saccade latency in C homozygotes compared to T allele carriers in the patient group (*p* < 0.001). In the control group, however, the effect of the *GAD1* genotype was not significant (*p* = 0.82) (Fig. 3b).

The coefficient of variation for the latency of correct saccades was not dependent on the genotype [*F*(1, 116) = 0.16, *p* = 0.84], although an effect of group [*F*(1, 116) = 11.04, *p* < 0.01] but not a group x genotype interaction [*F*(1,1, 113) = 0.38, *p* = 0.69] was observed (Fig. 3c).

### Predictive logit-models with antisaccades

Overall, the results demonstrated that patients differed from controls in their error rate, antisaccade latency and coefficient of variation within the latencies for correct saccades. Subsequently, whether these measures could serve as predictors for group differentiation was evaluated using binary logistic regression, where group (*y* = 0 for healthy controls and *y* = 1 for schizophrenic patients) was taken as a dependent variable for different *COMT* and *GAD1* genotypes. This approach was fruitful only in the case of the *GAD1* genotypes.

#### Logit-regression analysis with variants of the *GAD1* genotype

The between-group difference in antisaccade latency was significant only in carriers of the CC variant of *rs3749034*. A comparison was then made between the predictive validities of the logistic regression model in the total sample, CC carriers and CT + TT carriers. Three equations were generated by STATISTICA 6.0 (Table 4), where ***y*** was the probability of the tested person attribution to the schizophrenic patient group and three sets of predictors (error rate, saccade latency and CV) were used as arguments.

**Table 4:** Results of logit analysis with error rate, latency and latency variability (CV) for correct saccades as predictors for differentiation of healthy subjects and schizophrenic patients in the total sample and in the samples with different genotypes of *GAD1*

Samples	Model quality	Specificity (correctly identified healthy subjects, %)	Sensitivity (correctly identified patients, %)	Accuracy (correctly classified participants in the total sample, %)	Odds ratio
Total	*χ* ^2^ = 47.2 *p* < 0.0001	90.6 (48 of 53)	67.7 (44 of 65)	78.0 (92 of 118)	17.5
CC carriers	*χ* ^2^ = 26.8 *p* < 0.0001	90.9 (20 of 22)	91.2 (31 of 34)	91.1 (51 of 56)	84.9
CT + TT carriers	*χ* ^2^ = 27.2 *p* = 0.0010	100% (31 of 31)	48.4 (15 of 34)	74.8 46 of 65	15.4

A relatively high level of specificity of correctly identified healthy subjects (90–100%) was observed in all three samples. At the same time, the sensitivities (correctly identified patients) of models in the total sample and in the CT + TT sample were comparatively low (67.7 and 48.4% respectively), whereas in the sample of CC carriers, the sensitivity of the model was high (91.2%).

### Correlations between antisaccade measures and PANSS scores

#### Correlations within the total sample

In the total patient sample, 9 significant correlations were found. Analysis revealed that as a whole, impaired AS performance correlated with increased PANSS scores (Table 5). Error rate correlated positively with P6 (suspiciousness/persecution), N5 (difficulty in abstract thinking) and O10 (disorientation) scales. AS latency correlated positively with N1 (blunted affect), N5, N6 (lack of spontaneity and flow of conversation), and O7 (motor retardation) scales, but negatively with the P4 scale (hyperactivity), and latency variability correlated positively with the N2 scale (emotional withdrawal).

**Table 5:** Correlations between antisaccade measures and PANSS scores in the total sample of patients carrying different *COMT* or *GAD1* genotypes

	**PANSS**	Total sample *n* = 65		*COMT*				*GAD1*			
				Met/Met + Val/Met *n* = 44		Val/Val *n* = 21		CC *n* = 34		*n* = 31 CT + TT	
		*r*	*p*	*r*	*p*	*r*	*p*	*r*	*p*	*r*	*p*
Error rate	P1 delusions	0.24	0.11	0.31	0.25	0.42	0.10	**0.47**	**0.026**	0.02	0.93
	P6 suspiciousness/persecution	**0.46**	**0.001**	0.32	0.12	**0.73**	**0.002**	**0.53**	**0.011**	**0.42**	**0.042**
	N5 difficulty in abstract thinking	**0.30**	**0.046**	0.25	0.27	**0.67**	**0.006**	0.24	0.27	0.36	0.085
	O10 disorientation	**0.35**	**0.031**	**0.31**	**0.049**	**0.44**	**0.049**	0.20	0.37	**0.42**	**0.041**
Latency	P4 hyperactivity	**0.32**	**0.031**	**−** 0.15	0.51	**− 0.69**	**0.006**	**−** 0.15	0.48	**− 0.61**	**0.002**
	N1 blunted affect	0.31	0.053	0.23	0.11	0.39	0.20	0.16	0.47	**0.47**	**0.021**
	N5 difficulty in abstract thinking	**0.32**	**0.049**	**0.40**	**0.026**	**−** 0.08	0.75	0.11	0.63	**0.39**	**0.038**
	N6 lack of spontaneity and flow of conversation	**0.33**	**0.024**	0.21	0.27	**0.64**	**0.011**	**−** 0.15	0.50	0.32	0.13
	O7 motor retardation	**0.45**	**0.002**	**0.34**	**0.043**	**0.69**	**0.005**	**0.44**	**0.043**	**0.46**	**0.023**
CV	N2 Emotional withdrawal	**0.33**	**0.027**	0.23	0.21	**0.51**	**0.036**	0.25	0.25	0.38	0.11

All values in bold text are significant (*p* < 0.05)

CV, coefficient of variation

#### Variants of the COMT genotype

In the case of Val homozygotes, correlations between AS measures and PANSS scores were very similar to those for the total sample, and only the correlation between AS latency and N5 scale was insignificant. In contrast, within the sample of Met carriers, only 3 correlations were significant. These were between error rate and O10, AS latency and N5, along with AS latency and the O7 score (Table 5).

#### Variants of the *GAD1* genotype

In the sample of T carriers, 6 significant correlations were detected. In comparison with the total sample, one new association between AS latency and blunted affect (N1) was unmasked, whilst 3 correlations from the total sample were insignificant (Table 4). Within the sample of C homozygotes, only 3 correlations were significant, namely, error rate which correlated positively with P1 (delusions), P6 scores (lack of spontaneity and flow of conversation), in addition to AS latency which correlated positively with O7 score (motor retardation).

In conclusion, error rate correlated predominantly with positive scales (P1, P6) and disorientation (O10). Latency correlated positively with negative scales (N1, N5, N6) and motor retardation (O7), and negatively with P4 (hyperactivity). Both error rate and AS latency correlated with difficulty in abstract thinking (N5) and only one positive correlation between latency variability (CV) and emotional withdrawal (N2) was obtained. The largest number of significant correlations was obtained for Val homozygotes (*rs4680*) and T carrier (*rs3749034*) samples.

## Discussion

**Fig. 4:**
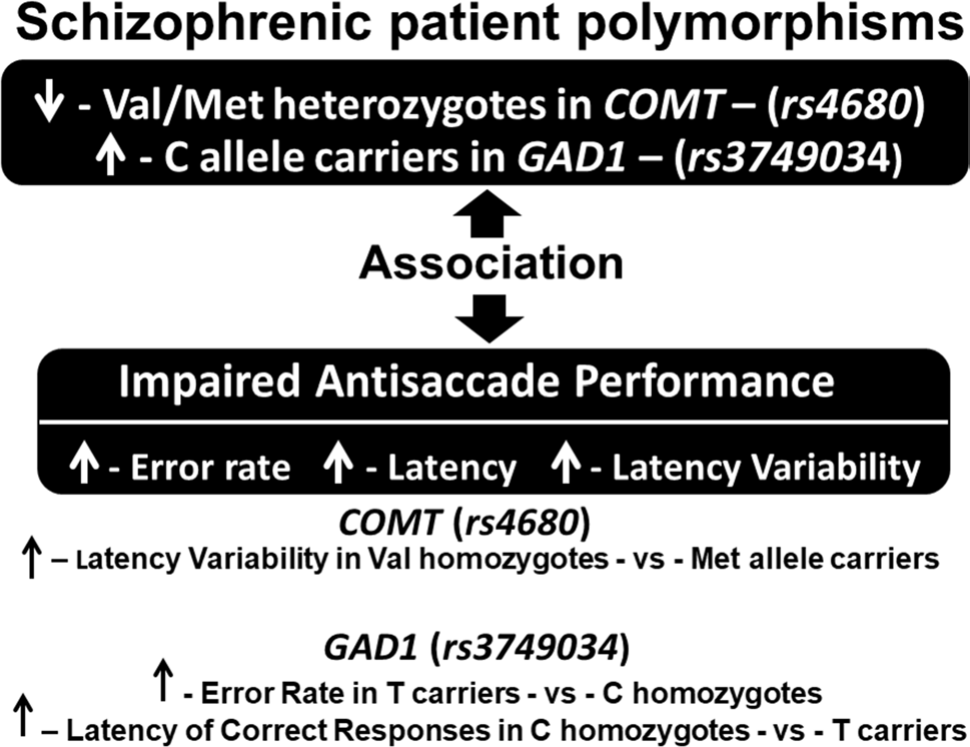
Scheme summarizing the principal findings

A possible association between *COMT* Val158Met and *GAD1 rs3749034* polymorphisms with antisaccade task (AS) performance was investigated in schizophrenic patients. In summary, the following principal findings were obtained: (1) genotyping revealed a decreased frequency of Val/Met heterozygotes in *COMT rs4680* polymorphism and an increased frequency of C allele carriers in *GAD1 rs3749034* polymorphism in patients compared to controls; (2) AS performance was impaired in schizophrenic patients versus controls, whereby patients displayed elevated error rates, and increased response latencies as well as latency variability (coefficient of variation) of correct responses compared to controls; (3) the polymorphisms in question were associated with the AS performance modifications in patients but not controls; (4) In the case of the *COMT rs4680* polymorphism, an augmented latency variability was observed in Val homozygotes compared to Met allele carriers in patients; (5) for the *GAD1 rs3749034* polymorphism, an elevated error rate was found in T carriers relative to C homozygotes, although an increased latency of correct responses was noted in C homozygotes relative to T carriers (Fig. 4).

### *COMT* Val158Met polymorphism and antisaccade performance

The finding that there was a lower frequency of Val/Met heterozygote carriers among schizophrenic patients compared to healthy subjects coincides with that of Hoenicka et al., [56]. The result also accords with the outcome of the meta-analysis performed by Costas et al., [57], in which a deficit of *rs4680* heterozygotes among male schizophrenic patients suggested a protective effect for heterozygosis. The current data also concur with the notion that an inverted U-shaped relationship in dopamine signaling at the molecular level in the dorsolateral prefrontal cortex arises from optimal levels of gene product in *COMT* Val/Met heterozygote carriers.

Analysis of the outcome of *COMT rs4680* polymorphism on AS performance divulged an increased coefficient of variation of latency (CV) for correct saccades in Val homozygotes compared to Met carriers in the pooled samples of patients and controls and in the patient group.

This increased antisaccade latency variability in schizophrenic patients corroborates previously reported studies involving not only schizophrenic patients, but also individuals with schizotypal traits [29, 58, 59]. A specific increase in latency variability but not mean latency of visually driven saccades in schizophrenic patients prompted the hypothesis that this deficit may originate from a distinct frontal cortical aberration in schizophrenia [58–60]. In non-human primates, the response latency in saccadic tasks correlates with the rise time of neuronal activity between the resting state and a threshold level in the frontal eye field (FEF) [61]. Thus, in extrapolating to schizophrenic patients, any increased latency variability may well have been related to a primary increased variability in neuronal activity rise time.

Considering the postulation that the *COMT* Val allele is associated with increased dopamine inactivation, this might conceivably result in reduced dopamine neurotransmission and a somewhat compromised prefrontal cortical function. It is, therefore, speculated that alterations in the fine-tuning of cortical DA-ergic pathways may be responsible for abnormal (disorganized) neuronal activity and a decreased signal/noise ratio in the prefrontal cortex would in turn lead to increased latency variability in the AS task.

In our study, the impact of *COMT rs4680* polymorphism on the clinical symptomatology of schizophrenia has also been exposed. Hence, a higher General Psychopathology Score and twice as many correlations between AS performance measures and PANSS scores were unveiled in Val- compared to Met-homozygotes.

Only in the Val homozygote sample did CV exhibit a positive correlation with N2 score (emotional withdrawal), while the only distinctive correlation in the sample of Met allele carriers was a positive association between AS latency and N5 scale (Difficulty in abstract thinking). These results are compatible with the data obtained by others who reported that the presence of the Val allele was positively associated with prefrontal activation during emotional processing, while the Met allele was associated with greater prefrontal activation during cognitive processing [37].

Despite there being a selection of findings to date, a range of investigations have generally identified inferior performance in tests of executive cognition associated with the *COMT* Val allele. Furthermore, associations between the *COMT rs4680* polymorphism and endophenotypes are consistently more evident in schizophrenic patients than in healthy controls [11]. It has also emerged that reduced volumes of frontal brain areas are associated with the Val/Val genotype in schizophrenic patients [39] as well as individuals at high risk of psychosis [38]. Meanwhile, there are divergent reports concerning the impact of *COMT rs4680* polymorphism on antisaccade performance. In this context, a larger number of Val158 alleles have been associated ostensibly with briefer and less variable AS latencies in schizophrenic patients [29], whilst others have shown an absence of any association between *rs4680* polymorphism and AS performance [27, 30]. The results discrepancies between previous reports and our study may have been derived from methodological differences. Essentially, the experimental paradigm of Haraldsson and coworkers, [29] for example, was more complex (4 peripheral locations) and no training session was employed. Consequently, even the control subjects in their study expressed considerably more errors (19–36%) than in our study (4.8%). Other differences are related to the participant samples, since both men and women participated in both cited studies, handedness was not considered, and patients were on stable treatments. Conversely, in our study, all subjects were right-handed males, and all of the patients were not under treatment. Data regarding the selective action of genes or gene-trait interactions have accumulated in recent years [52]. In fact, trait-relevant genes have different effects in respect of gender, psychological traits and other inherited or acquired individual characteristics. Thus, a more homogeneous sample of subjects inevitably facilitates detection of specific genetic effects.

### *GAD1 rs3749034* polymorphism and antisaccade performance

This is a preliminary report regarding the effects of *GAD1 rs3749034* polymorphism on antisaccade performance. Subsequent analysis of the frequency of different variants of the *rs3749034* polymorphism exposed a higher frequency of CC carriers in the schizophrenic patient group compared to healthy controls. This finding is in agreement with data obtained by some researchers [48, 49], but it is in contrast with the results of others [51]. Sources of these discrepancies, including such factors as the contribution of gender or ethnicity, are yet to be resolved. Nonetheless, it is noteworthy that we identified a deleterious effect of *rs3749034* on antisaccade performance only in schizophrenic patients, just as we observed in the case of the diminished performance with the Val158Met homozygotes (Fig. 4). Analysis of AS performance measures between samples revealed that the error rate was higher in T carriers, but AS latency was increased in CC homozygotes. Moreover, an increase in the latencies relative to control levels was found only in CC homozygotes. The correlation pattern between antisaccade measures and PANSS scores included predominantly negative ratings in T carriers, and positive scores in CC carriers. Also, there were considerably more correlations for T carriers.

Error rate in the AS task is one of the most compelling candidates for a schizophrenia endophenotype and numerous studies have consistently reported a greater number of inappropriate reflexive saccades to the target in different populations of schizophrenia patients [12, 26]. Increased error rate in patients is also thought to be associated with an impaired ability to inhibit intrinsic responses to redundant or irrelevant inputs [26].

Concerning the antisaccade response latency, most studies also report protracted latencies for correct antisaccades in patients with schizophrenia compared to controls [12, 22, 26, 62]. It is well-known that in healthy subjects, antisaccade response latencies are more prolonged than prosaccades and this can be attributed to additional cognitive processes that are invoked to perform the AS task correctly. Empirically, antisaccades entail an aptitude to restrain a reflexive stimulus-driven prosaccade to a peripheral visual target, then to achieve a coordinated transformation and initiate a voluntary oculomotor response towards the opposite side to the target [14]. On the other hand, studies of visually guided saccades in schizophrenia have reported both a prominence of anticipatory saccades as well as bimodal patient latencies which are either slower or match those of healthy controls [63, 64]. It has, therefore, been proposed that a deficit in prefrontal cortical processes in relation to the oculomotor system is associated with a predisposition to schizophrenia [65].

In this connection, the genotype carried by *rs3749034* patients is likely to determine prefrontal cortical insufficiency and any ensuing impaired AS task performance inherent in schizophrenia. In addition, genotypic variations in *GAD1* generally, may well modify cognitive function and conceivably related adverse changes in brain structure allometry. The elevated error rate in the AS task disclosed by T carriers is among the most typical disordered paradigms in schizophrenia [12, 26]. Therefore, although the T-allele has a protective function in healthy subjects, this genotype might contribute to the poor performance of frontally mediated cognitive tasks and the occurrence of associated negative symptoms in patients with schizophrenia. This deduction also coincides with the proposition that both variants of *rs3749034* can play their own specific roles in the pathological processes [51].

### Gene x trait interaction in schizophrenic patients

An interaction of genotype and diagnosis was found not only for *rs4680*, but also to a large degree for *rs3749034*. So, the effects of both polymorphisms were observed principally in patients but not in healthy controls. This is evidence of the polygenic nature of schizophrenia and a major role of epistatic interaction in the development of the disease and its symptomatology.

This gene x trait interaction may be useful in the development of diagnostic tools, and in the present investigation, it has been shown that the selective use of AS evaluation in the sample of CC carriers substantially improved the quality of the diagnostic model. This approach was applied in our previous investigation where the Met/Met variant of *rs4680* was explored with respect to the risk of schizophrenia [66]. The development of a complex model that includes a number of genes and different relevant neurophysiological tests is the focus of future studies.

Further investigations in this direction warrant larger samples that will allow application of factor analysis to assess the effects of the studied polymorphisms on the behavioral control and mechanisms of psychopathology.

## References

[1] Weinberger DR, Egan MF, Bertolino A, Callicott JH, Mattay VS, Lipska BK, Berman KF, Goldberg TE (2001). Prefrontal neurons and the genetics of schizophrenia. Biol Psychiatry.

[2] Manoach DS (2003). Prefrontal cortex dysfunction during working memory performance in schizophrenia: reconciling discrepant findings. Schizophr Res.

[3] Eisenberg DP, Berman KF (2010). Executive function, neural circuitry, and genetic mechanisms in schizophrenia. Neuropsychopharmacol.

[4] Goldman-Rakic S (1999). The physiological approach: functional architecture of working memory and disordered cognition in schizophrenia. Biol Psychiatry.

[5] Tan H-Y, Callicott JH, Weinberger DR (2009). Prefrontal cognitive systems in schizophrenia: towards human genetic brain mechanisms. Cogn Neuropsychiatry.

[6] Cannon TD, Huttunen MO, Lonnqvist J, Tuulio-Henriksson A, Pirkola T, Glahn D, Finkelstein J, Hietanen M, Kaprio J, Koskenvuo M (2000). The inheritance of neuropsychological dysfunction in twins discordant for schizophrenia. Am J Hum Genet.

[7] Toulopoulou T, Picchioni M, Rijsdijk F, Hua-Hall M, Ettinger U, Sham P, Murray R (2007). Substantial genetic overlap between neurocognition and schizophrenia: genetic modeling in twin samples. Arch Gen Psychiatry.

[8] Gottesman I, Gould T (2003). The endophenotype concept in psychiatry: etymology and strategic intentions. Am J Psychiatry.

[9] Meyer-Lindenberg A, Weinberger DR (2006). Intermediate phenotypes and genetic mechanisms of psychiatric disorders. Nat Rev Neurosci.

[10] Tan H-Y, Callicot JH, Weinberger DR (2008). Intermediate phenotypes in schizophrenia genetics redux: is it a no brainer?. Mol Psychiatry.

[11] Ira E, Zanoni M, Ruggeri M, Dazzan P, Tosato S (2013). COMT, neuropsychological function and brain structure in schizophrenia: a systematic review and neurobiological interpretation. J Psychiatry Neurosci.

[12] Hutton SB, Ettinger U (2006). The antisaccade task as a research tool in psychopathology: a critical review. Psychophysiology.

[13] Broerse A, Crawford TJ, den Boer JA (2001). Parsing cognition in schizophrenia using saccadic eye movements: a selective overview. Neuropsychologia.

[14] Levy DL, Mendell NR, Holzman PS (2004). The antisaccade task and neuropsychological tests of prefrontal cortical integrity in schizophrenia: empirical findings and interpretative considerations. World Psychiatry.

[15] Doricchi F, Perani D, Incoccia C, Grassi F, Cappa SF, Bettinardi V, Galati G, Pizzamiglio L, Fazio F (1997). Neural control of fast-regular saccades and antisaccades: an investigation using positron emission tomography. Exp Brain Res.

[16] Curtis CE, D’Esposito M (2003). Success and failure suppressing reflexive behavior. J Cogn Neurosci.

[17] Brown MR, Vilis T, Everling S (2007). Frontoparietal activation with preparation for antisaccades. J Neurophysiol.

[18] O’Driscoll GA, Alpert NM, Matthysse SW, Levy DL, Rauch SL, Holzman PS (1995). Functional neuroanatomy of antisaccade eye movements investigated with positron emission tomography. Proc Natl Acad Sci USA.

[19] McDowell JE, Dyckman KA, Austin BP, Clementz BA (2008). Neurophysiology and neuroanatomy of reflexive and volitional saccades: evidence from studies of humans. Brain Cogn.

[20] Everling S, Krappmann P, Flohr H (1997). Cortical potentials preceding pro- and antisaccades in man. Electroencephalogr. Clin Neurophysiol.

[21] Klein C, Heinks T, Andresen B, Berg P, Moritz S (2000). Impaired modulation of the saccadic contingent negative variation preceeding antisaccades in schizophrenia. Biol Psychiatry.

[22] Kirenskaya AV, Myamlin VV, Novototsky-Vlasov VY, Pletnikov MV, Kozlovskaya IB (2011). The contingent negative variation laterality and dynamics in antisaccade task in normal and unmedicated schizophrenic subjects. Span J Psychol.

[23] Amador N, Schlag-Rey M, Schlag J (2004). Primate antisaccade. II. Supplementary eye field neuronal activity predicts correct performance. J Neurophysiol.

[24] Johnston K, Everling S (2008). Neurophysiology and neuroanatomy of reflexive and voluntary saccades in nonhuman primates. Brain Cogn.

[25] Schlag-Rey M, Amador N, Sanchez H, Schlag J (1997). Antisaccade performance predicted by neuronal activity in the supplementary eye field. Nature.

[26] Turetsky BI, Calkins ME, Light GA, Olincy A, Radant AD, Swerdlow NR (2007). Neurophysiological endophenotypes of schizophrenia: the viability of selected candidate measures. Schizophr Bull.

[27] Demily C, Louchart-de-la-Chapelle S, Nkam I, Ramoz N, Denise P, Nicolas A, Savalle C, Thibaut F (2016). Does COMT val158met polymorphism influence P50 sensory gating, eye tracking or saccadic inhibition dysfunctions in schizophrenia?. Psychiatry Res.

[28] Ettinger U, Kumari V, Collier DA, Powell J, Luzi S, Michel TM, Zedomi O, Williams SC (2008). Catechol-O-methyltransferase (COMT) val158met genotype is associated with BOLD response as a function of task characteristic. Neuropsychopharmacol.

[29] Haraldsson HM, Ettinger U, Magnusdottir BB, Sigmundsson T, Sigurdsson E, Ingason A, Petursson H (2010). Catechol-O-methyltransferase Val158Met polymorphism and antisaccade eye movements in schizophrenia. Schizophr Bull.

[30] Kattoulas E, Evdokimidis I, Stefanis NC, Avramopoulos D, Stefanis CN, Smyrnis N (2010). Monitoring antisaccades: inter-individual differences in cognitive control and the influence of COMT and DRD4 genotype variations. Exp Brain Res.

[31] Dickinson D, Elvevåg B (2009). Genes, cognition and brain through a COMT lens. Neuroscience.

[32] Lin JR, Cai Y, Zhang Q, Zhang W, Nogales-Cadenas R, Zhang ZD (2016). Integrated post-GWAS analysis sheds new light on the disease mechanisms of schizophrenia. Genetics.

[33] Boyle EA, Li YI, Pritchard JK (2017). An expanded view of complex traits: from polygenic to omnigenic. Cell.

[34] Tunbridge EM, Harrison PJ, Weinberger DR (2006). Catechol-o-methyltransferase, cognition, and psychosis: Val158Met and beyond. Biol Psychiatry.

[35] Chen J, Lipska BK, Halim N, Ma QD, Matsumoto M, Melhem S, Kolachana BS, Hyde TM, Herman MM, Apud J, Egan MF, Kleinman JE, Weinberger DR (2004). Functional analysis of genetic variation in catechol-O-methyltransferase (COMT): effects on mRNA, protein, and enzyme activity in postmortem human brain. Am J Hum Genet.

[36] Meyer-Lindenberg A, Nichols T, Callicott H, Ding J, Kolachana B, Buckholtz J, Mattat VS, Egan M, Weinberger DR (2006). Impact of complex genetic variation in COMT on human brain function. Mol Psychiatry.

[37] Heinz A, Smolka MN (2006). The effects of catechol O-methyltransferase genotype on brain activation elicited by affective stimuli and cognitive tasks. Rev Neurosci.

[38] McIntosh AM, Baig BJ, Hall J, Job D, Whalley HC, Lymer GK, Moorhead TW, Owens DG, Miller P, Porteous D, Lawrie SM, Johnstone EC (2007). Relationship of catechol-O-methyltransferase variants to brain structure and function in a population at high risk of psychosis. Biol Psychiatry.

[39] Ohnishi T, Hashimoto R, Mori T, Nemoto K, Moriguchi Y, Iida H, Noguchi H, Nakabayashi T, Hori H, Ohmori M, Tsukue R, Anami K, Hirabayashi N, Harada S, Arima K, Saitoh O, Kunugi H (2006). The association between the Val158Met polymorphism of the catechol-O-methyl transferase gene and morphological abnormalities of the brain in chronic schizophrenia. Brain.

[40] Lew SE, Tseng KY (2014). Dopamine modulation of GABAergic function enables network stability and input selectivity for sustaining working memory in a computational model of the prefrontal cortex. Neuropsychopharmacology.

[41] Akbarian S, Huang HS (2006). Molecular and cellular mechanisms of altered GAD1/GAD67 expression in schizophrenia and related disorders. Brain Res Rev.

[42] Benes FM, Berretta S (2001). GABAergic interneurons: implications for understanding schizophrenia and bipolar disorder. Neuropsychopharmacology.

[43] Fung SJ, Webster MJ, Sivagnanasundaram S, Duncan C, Elashoff M, Weickert CS (2010). Expression of interneuron markers in the dorsolateral prefrontal cortex of the developing human and in schizophrenia. Am J Psychiatry.

[44] Hashimoto T, Arion D, Unger T, Maldonado-Avilés JG, Morris HM, Volk DW, Mirnics K, Lewis DA (2008). Alterations in GABA-related transcriptome in the dorsolateral prefrontal cortex of subjects with schizophrenia. Mol Psychiatry.

[45] Lewis DA, Hashimoto T, Volk DW (2005). Cortical inhibitory neurons and schizophrenia. Nat Rev Neurosci.

[46] Torrey EF, Barci BM, Webster MJ, Bartko JJ, Meador-Woodruff JH, Knable MB (2005). Neurochemical markers for schizophrenia, bipolar disorder, and major depression in postmortem brains. Biol Psychiatry.

[47] Volk DW, Sampson AR, Zhang Y, Edelson JR, Lewis DA (2016). Cortical GABA markers identify a molecular subtype of psychotic and bipolar disorders. Psychol Med.

[48] Addington AM, Gornick M, Duckworth J, Sporn A, Gogtay N, Bobb A, Greenstein D, Lenane M, Gochman P, Baker N, Balkissoon R, Vakkalanka RK, Weinberger DR, Rapoport JL, Straub RE (2005). GAD1 (2q31.1), which encodes glutamic acid decarboxylase (GAD67), is associated with childhood-onset schizophrenia and cortical gray matter volume loss. Mol Psychiatry.

[49] Brauns S, Gollub RL, Walton E, Hass J, Smolka MN, White T, Wassink TH, Calhoun VD, Ehrlich S (2013). Genetic variation in GAD1 is associated with cortical thickness in the parahippocampal gyrus. J Psychiatr Res.

[50] Straub RE, Lipska BK, Egan MF, Goldberg TE, Callicott JH, Mayhew MB, Vakkalanka RK, Kolachana BS, Kleinman JE, Weinberger DR (2007). Allelic variation in GAD1 (GAD67) is associated with schizophrenia and influences cortical function and gene expression. Mol Psychiatry.

[51] Lett TA, Kennedy JL, Radhu N, Dominguez LG, Chakravarty MM, Nazeri A, Farzan F, Walter H, Heinz A, Mulsant BH, Daskalakis ZJ, Voineskos AN (2016). Prefrontal white matter structure mediates the influence of GAD1 on working memory. Neuropsychopharmacology.

[52] De Young CG, Clark R (2012). The gene in its natural habitat: the importance of gene-trait interactions. Devel Psychopathol.

[53] Biederman J, Kim JW, Doyle AE, Mick E, Fagerness J, Smoller JW, Faraone SV (2008). Sexually dimorphic effects of four genes (COMT, SLC6A2, MAOA, SLC6A4) in genetic associations of ADHD: A preliminary study. Am J Med Genet B Neuropsychiatr Genet.

[54] Tucker DM, Williamson PA (1984). Asymmetric neural control system in human self-regulation. Psychol Review.

[55] Swerdlow NR, Light GA, Sprock J, Calkins ME, Green MF, Greenwood TA, Gur RE, Gur RC, Lazzeroni LC, Nuechterlein KH, Radant AD, Ray A, Seidman LJ, Siever LJ, Silverman JM, Stone WS, Sugar CA, Tsuang DW, Tsuang MT, Turetsky BI, Braff DL (2014). Deficient prepulse inhibition in schizophrenia detected by the multi-site COGS. Schizophr Res.

[56] Hoenicka J, Garrido E, Martínez I, Ponce G, Aragüés M, Rodríguez-Jiménez R, España-Serrano L, Alvira-Botero X, Santos JL, Rubio G, Jiménez-Arriero M, Palomo T (2010). Gender-specific COMT Val158Met polymorphism association in spanish schizophrenic patients. Am J Med Genet B Neuropsychiatr Genet.

[57] Costas J, Sanjuán J, Ramos-Ríos R, Paz E, Agra S, Ivorra JL, Páramo M, Brenlla J, Arrojo M (2011). Heterozygosity at catechol-O-methyltransferase Val158Met and schizophrenia: new data and meta-analysis. J Psychiatr Res.

[58] Smyrnis N, Evdokimidis I, Stefanis NC, Avramopoulos D, Constantinidis TS, Stavropoulos A, Stefanis CN (2003). Antisaccade performance of 1,273 men: effects of schizotypy, anxiety, and depression. J Abnorm Psychol.

[59] Smyrnis N, Karantinos T, Malogiannis I, Theleritis C, Mantas A, Stefanis NC, Hatzimanolis J, Evdokimidis I (2009). Larger variability of saccadic reaction times in schizophrenia patients. Psychiatry Res.

[60] Karantinos T, Tsoukas E, Mantas A, Kattoulas E, Stefanis NC, Evdokimidis I, Smyrnis N (2014). Increased intra-subject reaction time variability in the volitional control of movement in schizophrenia. Psychiatry Res.

[61] Schall JD (2002). The neural selection and control of saccades by the frontal eye field. Phil. Trans Roy Soc Lond B.

[62] Fukushima J, Morita N, Fukushima K, Chiba T, Tanaka S, Yamashita I (1990). Voluntary control of saccadic eye movements in patients with schizophrenic and affective disorders. J Psychiatr Res.

[63] Slavutskaya MV, Kirenskaya AV, Kozlovskaya IB (2005). Novototskiĭ-Vlasov VYu, Shul’govskiĭ VV. Hum Physiol.

[64] Gale HJ, Holzman PS (2000). A new look at reaction time in schizophrenia. Schizophr Res.

[65] Curtis CE, Calkins ME, Iacono WG (2001). Saccadic disinhibition in schizophrenia patients and their first-degree biological relatives. A parametric study of the effects of increasing inhibitory load. Exp Brain Res.

[66] Kirenskaya AV, Storozheva ZI, Kolobov VV, Sherstnev VV (2015). The acoustic startle response and polymorphism of the gene for catechol-O-methyltransferase in the norm and in schizophrenia. Neurochem J.

